# Use of the Plasma Spectrum RMS Signal for Arc-Welding Diagnostics

**DOI:** 10.3390/s90705263

**Published:** 2009-07-03

**Authors:** Jesus Mirapeix, Adolfo Cobo, Jose Fuentes, Marta Davila, Juan Maria Etayo, Jose-Miguel Lopez-Higuera

**Affiliations:** 1 Photonics Engineering Group, Universidad de Cantabria, Santander E39005, Spain; E-Mails: adolfo.cobo@unican.es (A.C.); miguel.lopezhiguera@unican.es (J.M.L.-H.); 2 ITP (Industria de Turbo Propulsores S.A), Parque Tecnológico de Bizcaia Kanala Bidea, Edificio 300, 48170 Zamudio (Bizkaia), Spain; E-Mails: jose.fuentes@itp.es (F.F.); marta.davila@itp.es (M.D.); 3 ROBOTIKER-Tecnalia. Parque Tecnológico Edif. 202, 48170 Zamudio (Bizkaia), Spain; E-mail: etayo@robotiker.es

**Keywords:** plasma spectroscopy, on-line monitoring, arc-welding, fiber sensor, RMS signal

## Abstract

A new spectroscopic parameter is used in this paper for on-line arc-welding quality monitoring. Plasma spectroscopy applied to welding diagnostics has typically relied on the estimation of the plasma electronic temperature, as there is a known correlation between this parameter and the quality of the seams. However, the practical use of this parameter gives rise to some uncertainties that could provoke ambiguous results. For an efficient on-line welding monitoring system, it is essential to prevent the appearance of false alarms, as well as to detect all the possible defects. In this regard, we propose the use of the root mean square signal of the welding plasma spectra, as this parameter will be proven to exhibit a good correlation with the quality of the resulting seams. Results corresponding to several arc-welding field tests performed on Inconel and titanium specimens will be discussed and compared to non-destructive evaluation techniques.

## 1. Introduction

The on-line monitoring of both laser and arc-welding processes is nowadays, not only an active area of research, but also a hot topic in terms of the interest of the industry in its possible implementation for different specific applications. It is well known that the physics of these processes is of great complexity, which has also given rise to an important line of research [1–6]. Although there are sophisticated models dealing with the different process taking place within the welding plasma, most of these solutions cannot be used for real-time analysis, as they imply a significant computational cost [7]. It is precisely this complexity, in addition to the various external variables that can have an effect upon the process, what makes it impossible to theoretically relate the process input parameters to the final seam quality. The common approach in this context is to carry out extensive weld trials prior to the final configuration of the production setup. When the dynamics of the particular process for a specific material has been understood, and the optimal process parameters minimizing the appearance of defects determined, the production stage can take place. However, some defects may appear due to issues such as lack of cleanliness, misalignments, variations on the distance between the electrode tip and the seam, perturbations on the shielding gas flow, among others. To verify that the welding process has been performed following the specific quality requirements, non-destructive evaluation techniques (NDT) such as magnetic particles, leakage tests, ultrasound inspection or infrared thermography are performed on the welded specimens. In some cases destructive evaluation, such as macrographs or fatigue tests also have to be used.

With this framework it seems clear that a reliable and efficient on-line monitoring system would be of great interest for the industry, especially in those fields where quality requirements are more demanding, like aeronautics or the fabrication of heavy components for nuclear power stations. In spite of the great research effort in this field, the presence of commercial monitoring systems in production stages is still sparse, probably due to both resistance from the companies and lack of reliable systems able to work in industrial scenarios. Different approaches have been considered to face this problem, from sensors monitoring both voltage and current [8,9], to solutions based on the processing of the acoustic emission generated during the process [10,11], machine vision [12,13] or infrared thermography [14]. A natural approach in this regard lies in optical sensors able to capture the plasma radiation generated during the process. The subsequent processing can be carried out by using simple configurations based on photodiodes [15], or a more demanding but sophisticated analysis within the framework of plasma optical spectroscopy [16–18]. Given that the plasma reflects the events occurring during the process, a detailed analysis of the plasma spectra can produce monitoring parameters able to indicate the appearance of weld defects.

The plasma electronic temperature *T_e_* is the most common spectroscopic parameter employed to obtain a correlation with the quality of the seams [16–18], and it can be calculated by means of the Boltzmann-plot, where several emission lines from the same species are considered, or using a simplified equation involving only two emission lines. Although the correlation between this parameter and the appearance of weld defects is well known, there are some uncertainties that can lead to false alarms or undetected defects. The selection of the emission lines participating in the *T_e_* estimation is critical in this regard, and depending on the chosen instrumentation, the identification of those lines might involve some errors. It is worth mentioning that low-cost CCD spectrometers are typically selected in this regard, and the limitations in their spectral resolution gives rise to the issues mentioned above. Some processing strategies can be taken into account to improve this resolution [18], but it is also common to have problems in the number of emission lines to be found in the plasma spectra, as they have to fulfill an established criterion to reduce the uncertainty in the determination of *T_e_*. In this regard, we have performed studies to provide alternative processing solutions within the framework of plasma optical spectroscopy. In a previous paper a new monitoring parameter based on the calculation of the wavelength associated with the maximum intensity of the plasma continuum signal was proposed [19]. It should be mentioned that different processing schemes have also been proposed, based on correlation analysis [20] and synthetic spectra and optimization algorithms [21], but they, although based on the acquisition and analysis of the welding plasma spectra, leave aside the classical spectroscopic approach.

In this paper we propose the use of the plasma spectrum root mean square RMS signal as an alternative on-line monitoring parameter. A similar approach has been initially explored by Wang *et al*. [22] for laser welding of titanium alloys by using a photodiode. In our system a CCD spectrometer is employed, and the plasma spectrum RMS signal is calculated by considering the intensity associated with all the pixels in the sensor. With this approach it is possible to provide in real-time different spectroscopic monitoring parameters and, depending on the particular process, to use only one or to combine some of them under specific logic rules. Experimental arc-welding tests performed in the facilities of ITP (Industria de Turbo Propulsores S.A.), a company devoted to the fabrication of components for aeronautics, with both Inconel 718 and Titanium 6Al-4V specimens, will show the feasibility of the proposed solution. Results of visual and X-ray inspection of the seams and the possibility of classifying the different weld defects in terms of the spectroscopic parameters will be also discussed.

## 2. Plasma Diagnostics Applied to On-Line Welding Monitoring

As commented in the previous section, different spectroscopic monitoring parameters will be considered for the experimental analysis of the field tests. First of all, the plasma electronic temperature *T_e_* can be determined by means of the Boltzmann-plot, which is derived from the Boltzmann equation [23]:

(1) $$\text{ln}\left(\frac{I_{\mathrm{mn}}\;\lambda_{\mathrm{mn}}}{A_{\mathrm{mn}}\;g_{m}}\right)=\text{ln}\left(\frac{\mathrm{hcN}}{Z}\right)-\frac{E_{m}}{{\mathrm{kT}}_{e}}$$

where *E_m_* is the upper level energy, g_m_ the statistical weight, *A* the transition probability, *λ_mn_* the wavelength, *I_mn_* the emission line relative intensity, *k* the Boltzmann constant, *h* the Planck’s constant, *c* the light velocity, *N* the total population density of the element and *Z* the partition function. The representation of the left-hand side of Equation (1) versus *E_m_* has a slope inversely proportional to *T_e_*. Several emission lines from the same species are considered in this case to obtain the *T_e_* profile, but this can be simplified by choosing only two lines and using Equation (2):

(2) $$T_{e}=\frac{E_{m}(2)-E_{m}(1)}{k\text{ln}\left[\frac{I(1)A(2)g_{m}(2)\lambda (1)}{I(2)A(1)g_{m}(1)\lambda (2)}\right]}$$

Equation (2) is commonly employed for on-line welding monitoring, given its reduced computational cost. However, it is worth mentioning that the temperature profiles will be noisier with this approach, what can be a problem for this kind of application.

As mentioned in Section 1, the wavelength associated with the maximum intensity of the plasma continuum *λ_max_* was proposed for monitoring purposes in a previous paper [19]. To obtain this parameter an initial stage based on a smoothing algorithm or a low-pass filter is required to remove the contribution of the emission lines in the plasma spectra. The resulting background signal is then modeled as a blackbody radiation via the Planck function:

(3) $$B_{\lambda }\;(T)=\frac{{2\mathrm{hc}}^{2}}{\lambda^{5}}\frac{1}{\left(e^{\frac{\mathrm{hc}}{\mathrm{kT}\lambda }}-1\right)}$$

In Equation (5)*T* is the temperature that has to be previously obtained by means of:

(4) $$\lambda_{\text{max}}=\frac{2.9\cdot 10^{6}}{T}$$

where *λ_max_* (nm) is the wavelength associated with the maximum intensity of the plasma spectrum after the filtering process.

**Figure 1.**
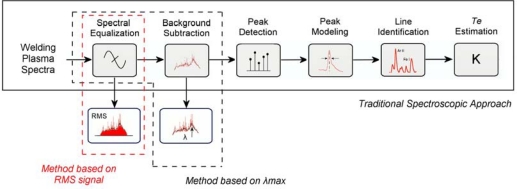
Block diagram of the processing stages involved in the estimation of the monitoring parameters.

The RMS signal of the plasma spectrum is calculated by considering the intensity provided by each pixel of the CCD sensor included in the setup, without considering the background subtraction stage:

(5) $$S_{\mathrm{RMS}}=\sqrt{\frac{1}{n}\sum_{i=0}^{n-1}x_{i}^{2}},$$

where *n* is the total number of pixels of the CCD and *x_i_* the intensity corresponding to the i-th pixel. This signal indicates an estimation of the average intensity of the radiation emitted by the welding plasma, and, consequently, it is a indirect measurement of the plasma spectrum energy within the spectral range considered, and it is also related to the total arc power, and consequently to the process heat input. Typical estimations indicate that approximately 20% of the total column energy is lost by radiation in a TIG arc at 100 A [24]. Figure 1 depicts the processing stages required for each monitoring parameter, thus showing an indication of their computational cost.

## 3. Experimental Issues and Results

The arc-welding tests were performed in the facilities of Industria de Turbo Propulsores, S.A. (ITP), in Zamudio (Spain). These tests consisted of 29 seams performed on Inconel 718 plates (2 mm thickness) and 24 on Titanium 6Al-4V (1.6 mm thickness), where different defective situations were examined. Table 1 shows the relevant composition of these two materials. The arc-welding process was a pulsed TIG (Tungsten Inert Gas) with Ar acting as shielding gas. The optimal values for the Inconel tests were 115 and 45 A for the high and low currents, a welding speed of 180 mm/min, a filler wire speed of 700 mm/min, a shielding gas flow rate of 10 L/min and a stick-out (distance between the electrode tip and the plate) of 1.08 mm. In the case of titanium these values were 75 and 35 A, a welding speed of 120 mm/min, a shielding gas flow rate of 10 L/min and a stick-out of 1.08 mm. The tests with titanium were performed without filler wire, and in both cases the shielding gas was also guided to the bottom side of the plates with a flow rate of 30 L/min.

The optical monitoring system used during the field tests was formed by a 600 μm core fiber of 10 m length attached to a bifurcated optical fiber (an Y-shaped assembly with two 600 μm core fibers side-by-side in the common end). The end of the fiber used as input optics of the system was placed at approximately 10 cm from the electrode tip. This arrangement was used to simultaneously provide the plasma radiation to two different CCD spectrometers (Ocean Optics USB2000 and HR4000) with wavelength ranges of 195 to 535 and 370 to 500 nm respectively. The RMS signal is calculated separately for each spectrometer, thus allowing comparison of the obtained results. The whole spectral range was considered to generate the plasma RMS profiles with both spectrometers. It should be mentioned that no additional input optics, i.e., optical collimator, was attached to the SMA connector of the fiber located at the end of the weld torch and focused on the plasma column. The acquisition and analysis of the plasma spectra was accomplished by means of a laptop computer.

### 3.1. Tests on Inconel 718

**Figure 2.**
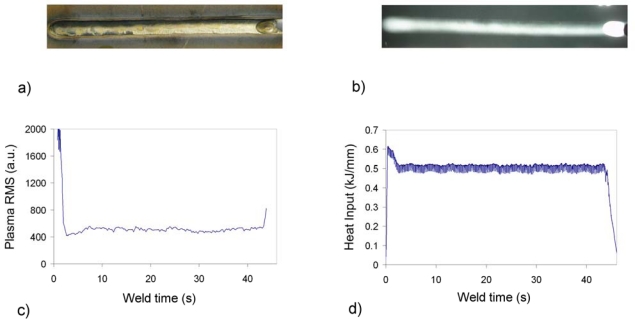
Plasma RMS and heat input profiles for weld test free of defects on Inconel 718.

Results corresponding to a seam free of defects are shown in Figure 2. Figure 2(a),(b) shows the bottom and X-ray images of the seam. The visual, X-ray and penetrant liquids inspections conclude that the seam is acceptable, only showing a light sinkage. Figure 2(c),(d) depicts the corresponding plasma RMS determined with spectrometer USB2000 and heat input profiles, the latter determined as the product between the RMS values of both voltage and current of the process, and divided by the torch speed (these data have been provided by ROBOTIKER-Tecnalia). Given that this speed can be considered constant through all the performed tests, there is a direct correlation between this parameter and the arc power, and therefore, it can be interesting to compare it with the plasma RMS signal. As expected for a seam free of defects, the profiles depicted in Figure 2(c),(d) does not show strong perturbations that can be associated with weld defects.

**Figure 3.**
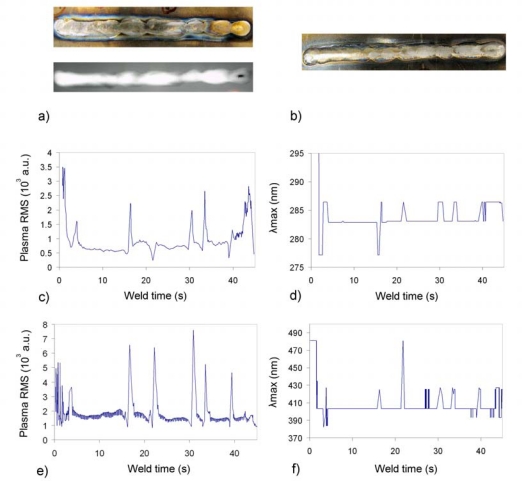
Plasma RMS and *λ_max_* profiles for defective weld on Inconel 718.

The seam presented in Figure 3(a),(b) (top and X-ray, and bottom, respectively) exhibits clear irregularities. The case study was to produce a burn-trough by increasing the welding current. The resulting seam was classified as non-acceptable due to both contamination and a concavity of 0.3 mm, representing a 15% in terms of the plate thickness. Some of the seam irregularities can be correlated with the perturbations on the plasma RMS and *λ_max_* profiles. The profiles of Figure 3(c),(d) was determined with data from the USB2000 spectrometer (spectral range from 195 to 535 nm, optical resolution of 0.3 nm), while the ones in Figure 3(e),(f) is associated with the HR4000 spectrometer (spectral range from 370 to 500 nm, optical resolution of 0.03 nm). The interest of this comparison lies mainly in the *λ_max_* profiles, as the different spectral ranges are supposed to affect their final response. However, it can be appreciated the similarity between both signals, differing mainly in the sharp dip of Figure 3(d) at x ≈ 16 s, and the peaks to be found in Figure 3(f) at x ≈ 27 s.

In terms of the plasma RMS signals, the perturbations are quite similar in both situations, although the peaks in Figure 3(e) at approximately 22 and 39.5 s appear as dips in Figure 3(a). This can be explained mainly by the higher acquisition rate of HR4000, which is able to follow fast variations of the plasma radiation. Typical acquisition rates with our setup are in the order of 80 spectra per second for USB2000, and approximately 200 for HR4000. The noisier profile obtained in Figure 3(e) is also due to this issue, as the effect of the welding current pulses can be observed in this case. The number of spectral samples captured for this test were 241 and 977 for USB2000 and HR4000, and the average integration times used for both spectrometers during the process were 152 and 30 ms, respectively. In this regard, it is worth noting that for this particular scenario the acquisition rate of the USB2000 seems to be good enough, but for faster welding processes, like laser-welding, it could prove to be inadequate.

**Figure 4.**
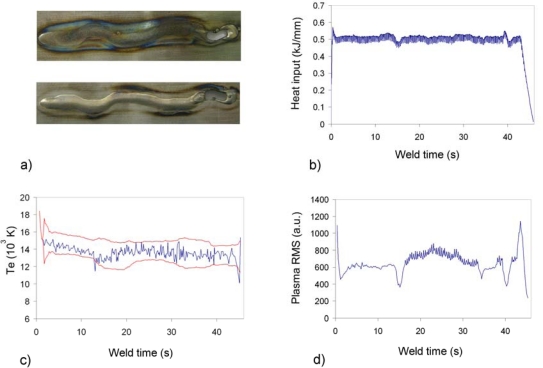
Heat input, *T_e_* and plasma RMS profiles for defective weld (trajectory deviation) on Inconel 718.

A deviation in the welding head trajectory was provoked to analyze the monitoring signals ability to detect this kind of defect. Figure 4(c),(d) was obtained with data captured by USB2000. By matching Figure 4(a) and (b), it can be concluded that the heat input profile detects a defect at x ≈ 15 s, but the remaining profile does not exhibit any other clear perturbation. The *T_e_* profile determined by means of two Ar II emission lines (with NIST [25] wavelengths at 460.96 and 487.99 nm) presents a similar response in this regard, raising an alarm at x ≈ 13 s. It should be mentioned that the automatic defect detection algorithm is based on the proposal by Ancona *et al*. [26]. Consequently, the thresholds of Figure 4(c) are generated by considering the *T_e_* mean and standard deviation values, and also by including in the model two main parameters allowing to regulate the threshold separation from the *T_e_* mean value and the window size determining the number of CCD pixels included in each calculation. This model has been slightly modified by removing from the analysis those temperature values exceeding a pre-established limit, and also the higher temperatures within the window size. Although this approach has been initially applied to the *T_e_*, it could be also extended to the plasma RMS and *λ_max_* profiles for practical considerations. The result depicted in Figure 4(d) indicates a higher sensitivity to the studied defect, considering not only the perturbations at x ≈ 15, x ≈ 34.5 and x ≈ 40 s, but also the “ripple signal” to be observed between x ≈ 15 and x ≈ 34.5 s. We believed that this behaviour of the plasma RMS signal is related to the defective situation, and probably reflects oscillations of the weld pool.

**Figure 5.**
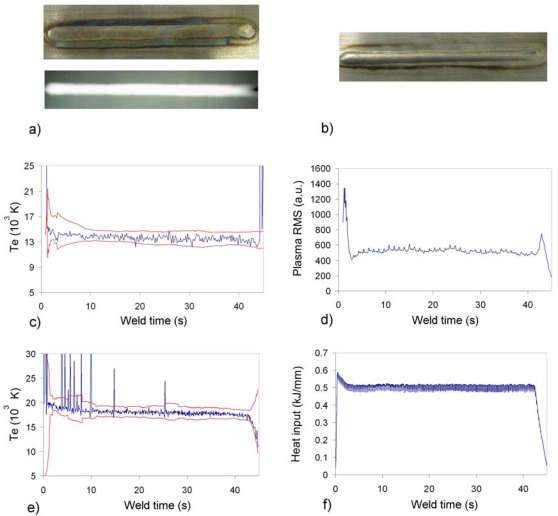
Heat input, *T_e_* and plasma RMS profiles for defective weld (misaligment) on Inconel 718.

Another interesting example in this regard is displayed in Figure 5, the defect in this case being a misalignment between the two plates. The measured misalignment was 1 mm, greatly exceeding the established threshold of 0.3 mm. The heat input profile displayed in the figure does not allow detection of this particular defect, as the signal remains unaltered through the whole process. The same happens with the plasma RMS signal (USB2000), although in this case, the “ripple signal” mentioned above appears again, and, with the suitable processing, could be used for monitoring purposes. Temperature profiles for spectrometers USB2000 and HR4000 are presented in Figures 5(c) and (d). In this case there are clear differences between both signals, appearing in the latter clear deviations from the *T_e_* mean value. The discrepancies between the average estimations of *T_e_* in this case are related to the simple approach used for the background subtraction process, where a smoothing algorithm with a moving window of 50 spectral samples was employed, and the poor resolution involved in the equalization of the CCD response.

### 3.2. Tests on Titanium 6Al-4V

**Figure 6.**
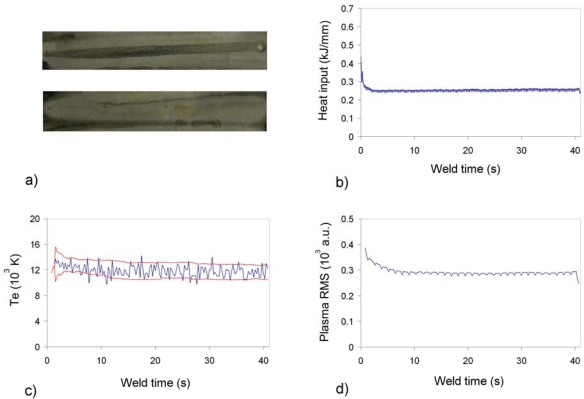
Heat input, *T_e_* and plasma RMS profiles for defective weld (lack of penetration) on Titanium 6Al-4V.

Results regarding a correct seam will not be displayed again for the titanium tests, given that the profiles show constant signals with absence of relevant perturbations. In Figure 6(a) new defective situation, lack of penetration, is studied. As expected given the reduction on the welding current (45 and 15 A) the mean values of the different signals exhibit a clear decrease. *T_e_* and RMS profiles were calculated with data from USB2000.

**Figure 7.**
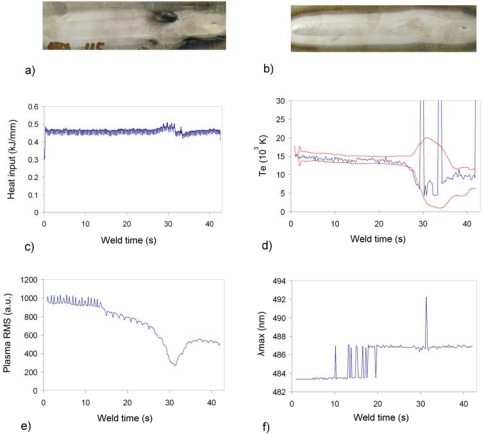
Heat input, *T_e_*, plasma RMS and *λ_max_* profiles for defective weld (presence of oil) on Titanium 6Al-4V.

**Figure 8.**
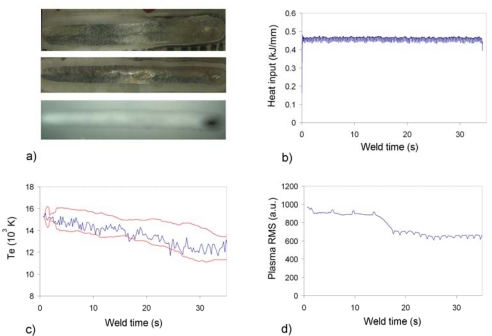

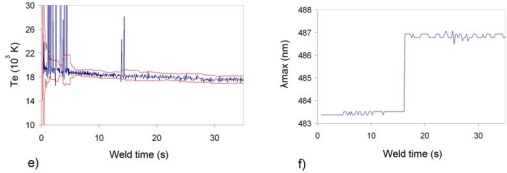
Heat input, *T_e_*, plasma RMS and *λ_max_* profiles for defective weld (presence of oil) on Titanium 6Al-4V.

The defect that can be seen in Figure 7 was produced by the application of oil over the plate interface before the welding process. In this way, a situation of lack of cleanliness was simulated, and the resulting flaw at x ≈ 30 s generated. There is a clear correlation among the heat input, *T_e_*, *λ_max_* and plasma RMS profiles obtained with USB2000, all of them indicating an event in the defect location. However, it is worth noting that the plasma RMS signal starts to decrease at x ≈ 15 s, thus showing a possible defective situation, probably due to the presence of oil in that position. A similar example is presented in Figure 8, but in this case there is no defect to be seen in the seam and, in addition, both visual and X-ray inspection conclude that the seam was correct. However, all the spectroscopic parameters indicate a clear deviation (Figure 8(c),(d),(f) obtained with USB2000; Figure 8(e) with HR4000), although the heat input profile is not in agreement with this alarm. In this regard, further destructive analysis will be performed on this plate to try to clarify this lack of correlation among the different techniques.

## 4. Conclusions

Application of a new spectroscopic parameter, the plasma RMS signal, has been proposed for the real-time monitoring of arc-welding processes. This signal can be generated with a reduced computational cost in comparison to the traditional approach in this field, consisting on the estimation of the plasma electronic temperature *T_e_*. This new technique has been checked by means of several field tests on both Inconel 718 and Titanium 6Al-4V in the facilities of ITP (Industria de Turbo Propulsores), where typical defects have been provoked and then inspected by means of visual and X-ray inspection. The spectroscopic system was formed by two different spectrometers, thus allowing a more detailed analysis and comparison of the resulting signals, especially of *T_e_*. As an additional validation, all the spectroscopic parameters have been also compared to heat input profiles provided by an alternative monitoring system by ROBOTIKER-Tecnalia. The proposed parameter is directly related to the arc power, but it has been demonstrated that, in some particular scenarios, it offers more information for given defects than the associated heat input profiles. The combination of the different spectroscopic parameters might give rise to an attempt of defect classification, as the plasma RMS signal has proved to be useful in detecting defects such as misalignment or trajectory deviation that can be otherwise difficult to be identified.

Further field tests will be conducted to investigate the appearance of the so-called “ripple signal” on the plasma RMS profile, and to conclude, by means of destructive evaluation, whether the ambiguous plates that have been labeled as defective by the spectroscopic monitoring system really exhibit any defects. In addition, an analysis of the suitability of using spectral windows within the spectrometer range to generate the RMS profile will be also conducted, what would imply a further improvement in the computational performance of the system. Alternative approaches for the background subtraction process, a better estimation of the spectrometer spectral response and the effect of the elimination of the plasma background signal before determining the RMS profiles will be also investigated.

## Figures and Tables

**Table 1.** Composition of Inconel 718 and Titanium 6Al-4V.

**Inconel 718**		**Titanium 6Al-4V**	

**Element**	**% participation**	**Element**	**% participation**
Fe	Balanced	Ti	Balanced
Ni (+Co)	50–55	Al	5.5–6.75
Cr	17–21	V	3.5–4.5
Mo	2.8–3.3	C	0.08 max
Nb (+Ta)	4.75–5.5	H	0.015 max
Ti	0.65–1.15	Fe	0.25 max
Al	0.2–0.8	N	0.05 max
Si	0.35	O	0.2 max
Mn	0.35		

## References

[1] Eagar T.W., North T.H. (1990). Physics of arc welding processes. Advanced Joining Technologies.

[2] Wu C.S., Ushio M., Tanaka M. (1997). Analysis of the TIG welding arc behaviour. Computat. Mater. Sci.

[3] Haidar J. (1998). A theoretical model for gas metal arc welding and gas tungsten arc welding. I. J. Appl. Phys. (USA).

[4] Xu G., Hu J., Tsai H.L. (2008). Three-dimensional modeling of the plasma arc in arc welding. J. Appl. Phys. (USA).

[5] Dowden J., Kapadia P. (1994). Plasma arc welding: a mathematical model of the arc. J. Appl. Phys. (USA).

[6] Tanaka M., Lowke J.J. (2007). Predictions of weld pool profiles using plasma physics. J. Phys. D. Appl. Phys. (UK).

[7] Gornushkin I.B., Kazakov A.Y., Omenetto N., Smith B.W., Winefordner J.D. (2005). Experimental verification of a radiative model of laser-induced plasma expanding into vacuum. Spectrochim. Acta Part B.

[8] Li L., Brookfield D.J., Steen W.M. (1996). Plasma charge sensor for in-process, non-contact monitoring of the laser welding process. Meas. Sci. Technol.

[9] Lu W., Zhang Y.M., Emmerson J. (2004). Sensing of weld pool surface using non-transferred plasma charge sensor. Meas. Sci. Technol.

[10] Gu H., Duley W.W. (1996). Statistical approach to acoustic monitoring of laser welding. J. Phys. D.

[11] Farson D.F., Kim K.R. (1999). Generation of optical and acoustic emissions in laser weld plumes. J. Appl. Phys.

[12] Zhang G.J., Yan Z.H., Wu L. (1997). Visual sensing of weld pool in variable polarity TIG welding of aluminium alloy. Trans. Nonferr. Met. Soc. China.

[13] Kovacevic R., Zhang Y.M., Ruan S. (1995). Sensing and control of weld pool geometry for automated GTA welding. J. Eng. Ind. Trans. ASME.

[14] Wikle H.C., Kottilingam S., Zee R.H., Chin B.A. (2001). Infrared sensing techniques for penetration depth control of the submerged arc welding process. J. Mat. Proc. Technol.

[15] Gu H., Duley W.W. (1995). Possible diagnostic signal for monitoring CO_2_ laser welding of aluminum alloy sheets. Proc. SPIE.

[16] Sforza P., de Blasiis D. (2002). On-line optical monitoring system for arc welding. NDT E Int.

[17] Ancona A., Spagnolo V., Lugara P.M., Ferrara M. (2001). Optical sensor for real-time monitoring of CO_2_ laser welding process. Appl. Opt.

[18] Mirapeix J., Cobo A., Conde O.M., Jaúregui C., López-Higuera J.M. (2006). Fast algorithm for spectral processing with application to on-line welding quality assurance. Meas. Sci. Technol.

[19] Mirapeix J., Cobo A., Fernandez S., Cardoso R., Lopez-Higuera J.M. (2006). Spectroscopic analysis of the plasma continuum radiation for on-line arc-welding defect detection. J. Phys. D.

[20] Sibillano T., Ancona A., Berardi V., Schingaro E., Parente P., Lugara P.M. (2006). Correlation spectroscopy as a tool for detecting losses of ligand elements in laser welding of aluminium alloys. Opt. Lasers Eng.

[21] Mirapeix J., Cobo A., González D.A., Lopez-Higuera J.M. (2007). Plasma spectroscopy analysis technique based on optimization algorithms and spectral synthesis. Opt. Expr.

[22] Wang C., Hu L., Hu X., Liu J. (2006). Relationship between plasma optic signal and penetration depth for partial-penetration laser welding. Chin. J. Mech. Eng. Engl. Ed.

[23] Griem H.R. (1997). Principles of Plasma Spectroscopy.

[24] Lancaster J.F. (1984). The physics of welding. Phys. Technol.

[25] National Institute for Standars and Technology (NIST) atomic spectra database, http://physics.nist.gov/cgi-bin/AtData/main_asd.

[26] Ancona A., Maggipinto T., Spagnolo V., Ferrara M., Lugara P.M. (2002). Optical sensor for real time weld defects detection. Proc. SPIE.

